# Pharmacologic Management of Coronary Artery Ectasia

**DOI:** 10.7759/cureus.17832

**Published:** 2021-09-08

**Authors:** Anwar Khedr, Bandana Neupane, Ekaterina Proskuriakova, Keji Jada, Sandrine Kakieu Djossi, Jihan A Mostafa

**Affiliations:** 1 Internal Medicine, California Institute of Behavioral Neurosciences & Psychology, Fairfield, USA; 2 Research, California Institute of Behavioral Neurosciences & Psychology, Fairfield, USA; 3 Psychiatry, Research, California Institute of Behavioral Neurosciences & Psychology, Fairfield, USA

**Keywords:** aneurysmal coronary artery disease, warfarin therapy, aspirin, ace inhibitors, statins, b-blockers, ca channel blockers, nitrates, trimetazidine, coronary artery ectasia (cae)

## Abstract

Coronary artery ectasia (CAE) is a rare form of aneurysmal coronary heart disease. It is defined as a dilatation of the coronary artery by more than one-third of its length and with a diameter 1.5 times of a normal coronary artery adjacent to it. This condition increases the risk of angina pectoris and acute coronary syndrome. Hence, we discuss the pharmacologic options for primary and secondary prevention of CAE complications. Antiplatelets such as aspirin are considered the mainstay of treatment in patients with CAE. Anticoagulants such as warfarin are warranted on a case-by-case basis to prevent thrombus formation depending on the presence of concomitant obstructive coronary artery disease and the patient’s risk of bleeding. Since atherosclerosis is the most common cause of CAE, statins are indicated in all patients for primary prevention. Angiotensin-converting enzyme (ACE) inhibitors may be indicated, especially in hypertensive patients, due to their anti-inflammatory properties. Beta-blockers may be indicated due to their antihypertensive and anti-ischemic effects. Calcium (Ca) channel blockers may be needed to prevent coronary vasospasm. Nitrates are generally contraindicated as they may lead to worsening of symptoms. Other antianginal medications such as trimetazidine can improve exercise tolerance with no reported adverse events in these patients.

## Introduction and background

**Figure 1:**
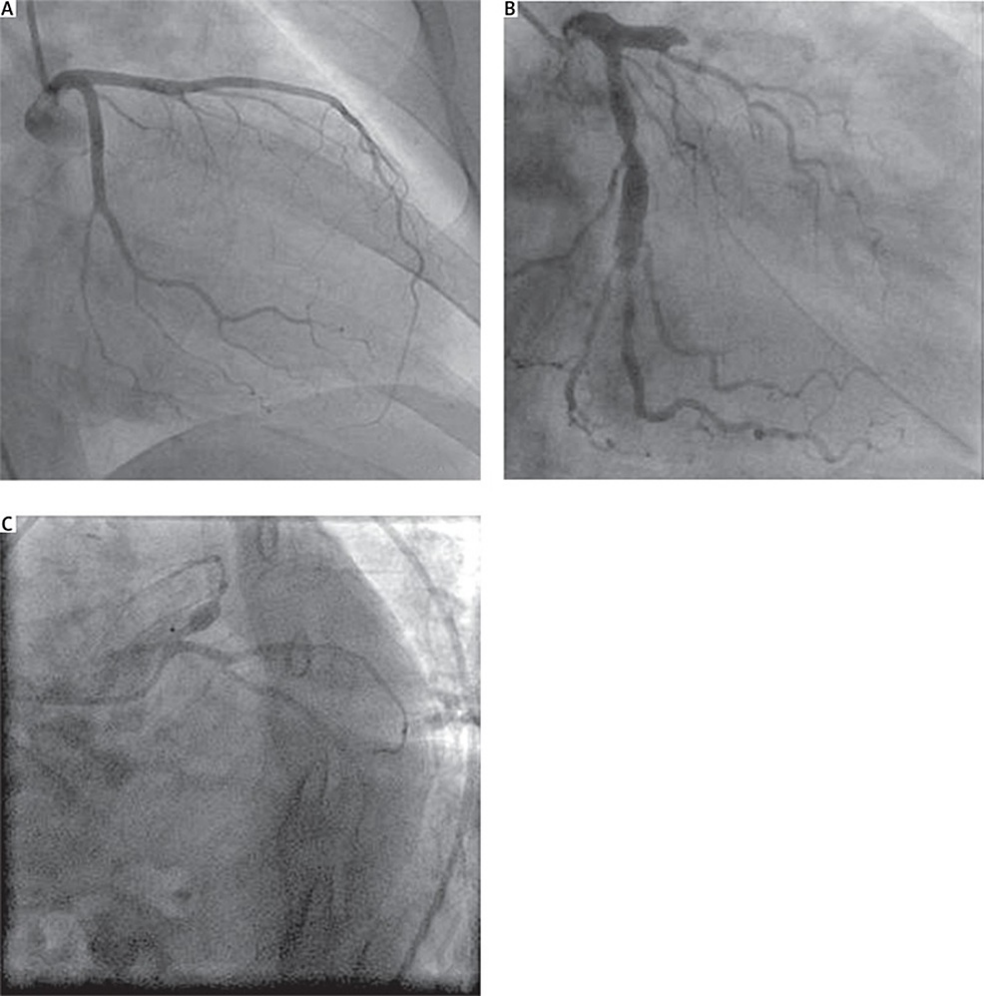
(A) Normal coronary artery; (B) Coronary artery ectasia; (C) Coronary artery aneurysm Copyright/License: Licensee Polish Journal of Thoracic and Cardiovascular Surgery, Poznań, Poland. This figure is from an open-access article distributed under the terms of the Creative Commons Attribution-NonCommercial-ShareAlike 4.0 International (CC BY-NC-SA 4.0) License. (https://creativecommons.org/licenses/by-nc-sa/4.0/). No modifications were made to the original figure.

Coronary artery ectasia (CAE) is a rare abnormal aneurysmal dilatation of the coronary arteries. Its prevalence ranges from 0.85 % to 5.3 % according to prior studies [1-3]. It is described as segmental dilation with a diameter 1.5 times that of the normal coronary artery next to it [4] and is differentiated from coronary artery aneurysms by its dilation that exceeds 1/3 the length of the coronary vessel, as shown in Figure 1 [5].

The right coronary artery is the most commonly affected in CAE [4]. Markis et al. classified CAE into four types according to the extent of the dilation and number of coronary arteries involved (Table 1) [6].

**Table 1:** Classification of coronary artery ectasia CAE: Coronary artery ectasia

CAE Type	Number of arteries involved	Extent of involvement
I	Two or more	Diffuse
II	Two	Diffuse in one vessel and discrete in another
III	One	Diffuse
IV	One	Discrete

Atherosclerosis is the most well-known cause of CAE. Congenital and collagen vascular disorders, infections, iatrogenic disease, and cardiac lymphomas among others are also causes [7]. Hypertension [4], smoking, male gender [8], and cocaine usage [9] are the most common risk factors. CAE's pathogenesis is yet unclear. The abnormal dilation of the lumen of the coronary arteries has been attributed to several processes. CAE is considered to be a type of excessive expansive remodeling that occurs in response to lytic enzymes' enzymatic breakdown of extracellular matrix, particularly matrix metalloproteinases, as well as tunica media thinning and chronic inflammation. High levels of homocysteine, C-reactive protein, and the vascular endothelial growth factor have been linked to CAE, suggesting that they may play a role in inflammation and neovascularization. According to another theory, increased nitric oxide (NO) levels can cause vasodilation and relaxation of ectatic areas [10]. Genetic factors such as angiotensin-converting enzyme DD genotype polymorphism [11], abnormal lipoprotein metabolism associated with familial hypercholesterolemia [12], potassium voltage-gated channel subfamily H member 1 (KCNH1) mutation [13], and autophagy related 16 like 1 (ATG16L1) gene mutations [14] have all been linked to CAE previously.

The most prevalent presenting symptom of CAE is angina pectoris [1]. Slow coronary flow can cause it even when there is no associated coronary artery stenosis. Acute coronary syndrome (ACS) can also be caused by distal embolization or the development of an occlusive thrombus. Complications such as rupture and the formation of shunts are possible [15]. For the diagnosis and assessment of CAE, coronary angiography is the investigation of choice [10]. In patients with isolated CAE, the degree of ectasia and backflow phenomenon in an ectatic left anterior descending artery were reported to be the most important angiographic markers of ischemia on exercise testing [16].

Despite the identification of this condition more than 50 years ago, its management is still a subject of debate due to insufficient evidence, and there are no uniform guidelines yet. The available management options are pharmacologic therapy, percutaneous intervention, and surgery [7]. In this review, we focus on the pharmacologic therapy options for CAE, their role in management, and the relation of this role to the pathogenesis, the clinical picture, and complications. Multiple options have been proposed to manage CAE and its complications, such as antiplatelet therapies, anticoagulants, lipid-lowering medications, angiotensin-converting enzyme (ACE) inhibitors, or angiotensin receptor blockers (ARBS), beta-blockers, calcium (Ca) channel blockers, and anti-anginal drugs. We will discuss the current literature on the use of each of these agents in CAE management and the indications of interventional management. We searched PubMed and Google Scholar and selected relevant articles. Keywords such as aneurysmal coronary artery disease, warfarin therapy, aspirin, ace inhibitors, statins, b-blockers, Ca channel blockers, nitrates, trimetazidine, and coronary artery ectasia, were searched individually or in combination to yield relevant information. We did not have any date or language restrictions due to the relatively low number of articles on the topic. Duplicated studies and studies providing insufficient and irrelevant information were excluded from our research.

## Review

Antiplatelets as the standard treatment

The exact pathogenesis of CAE remains unclear. Yasar et al. discovered that 32 patients with isolated CAE without associated coronary artery stenosis had significantly higher levels of P-selectin, beta-thromboglobulin, and platelet factor 4 (PF4) compared to a control group with normal coronary arteries (P<0.001), indicating increased platelet activation in these patients [17]. Moreover, a systematic review and meta-analysis study by Moghadam et al. found that patients with CAE had significant elevation of mean platelet volume compared to healthy individuals, suggesting that the thrombotic effects of platelets may be playing a role in the pathogenesis of CAE. Therefore, it is possible that antiplatelet therapies such as aspirin can play a role in the management of this disease [18].

The study by Swaye et al. did not find any significant increase in the incidence of myocardial infarction in patients with isolated CAE or any differences in the five-year survival rate compared to those without CAE. So, they suggested that CAE is just a variant of occlusive coronary atherosclerosis [4]. And that these patients should be placed routinely on aspirin as a tool of primary prevention [19].

The idea of adenosine diphosphate receptor inhibitors in combination with antiplatelet treatment has not yet been thoroughly studied in clinical trials. However, a systematic review of case reports by Pranata et al. concluded that the use of dual antiplatelet therapy is less effective than anticoagulants in preventing the recurrence of ACS in the context of CAE [20].

Anticoagulants for secondary prevention

Warfarin has been proposed as a treatment since ectatic coronary arteries are prone to thrombosis, dissection, and spasm [15, 21-22]. A case report by Perlman et al. proposed long-term warfarin treatment for patients with CAE. The study had a 41-year-old male patient with CAE who suffered from unstable angina and was found to have a partially occluding thrombus. The patient was administered heparin followed by warfarin, which resulted in the resolution of his symptoms, and he remained asymptomatic during a three-year follow-up [21].

In a study by Almazan et al., 23 patients with isolated CAE demonstrated the significant effects of warfarin by decreasing the incidence of unstable angina and positive exercise EKG, and also the duration of silent ischemia on EKG-Holter monitoring (P<0.001) [23]. In another study by Doi et al., CAE was associated with 3.25, 2.71, and 4.92-fold higher likelihoods of experiencing major adverse cardiac events (MACE), cardiac death, and non-fatal myocardial infarction respectively, after a 49-month follow-up. In addition, patients with CAE who received anticoagulant therapy and achieved optimal percentage of time in therapeutic range (%TTR) had no recurrences of MACE compared to those who did not receive any anticoagulant therapy or did not achieve %TTR (P=0.03) [24]. Pranata et al. also showed that patients with CAE who took anticoagulants were at a lower risk of ACS recurrence (P=0.035) [20].

In patients with isolated CAE, treatment with anticoagulants is still a matter of debate. In a study by Willner et al. 161 patients with CAE were followed for 10 years, and it was noted that the adverse clinical events risk was lower in patients with isolated CAE compared to patients who suffered from CAE with concomitant atherosclerotic coronary heart disease (P<0.05). So, they recommended against the use of anticoagulation therapy in patients with isolated CAE [2]. Their results supported the view of Demopoulos et al. that stated that patients with isolated CAE have a more favorable course and prognosis than CAE patients associated with obstructive coronary artery disease, and therefore deemed warfarin anticoagulation unnecessary [3]. However, Sorrel et al. proposed combining aspirin and warfarin for patients with CAE to prevent its potential complications. This regimen included aspirin 80 to 360mg/day to prevent platelet aggregation and warfarin while keeping the international normalized ratio (INR) between 2.0 and 2.5 to prevent thrombus formation. However, the study stated that the risks of any treatment should be weighed against the benefits in each patient to avoid the severe complication of bleeding [25]. Moreover, in an observational study by Grigoro, of the 20 patients with CAE type I, II, and III, 75 % of them were treated with warfarin plus aspirin 160mg/day. These patients were followed up for 4.2 years and were found to have a low percentage of adverse events and a 0% mortality rate [26]. However, Hart et al. suggested that long-term warfarin therapy should be implemented to decrease the risk of the formation of coronary thrombus and its deleterious consequences, while aspirin would be sufficient to manage asymptomatic CAE [27]. In Table 2, we summarize the studies that used warfarin for the management of CAE [20,23,24,26].

**Table 2:** Summary of the studies using warfarin for management of coronary artery ectasia CAE: Coronary artery ectasia; MACE: Major adverse cardiac events; ACS: Acute coronary syndrome; EKG: Electrocardiogram; %TTR: Percentage of time in therapeutic range

Authors/year of publication	Type of study	Intervention	Outcomes
Almazan et al. [23]./1997	Observational study	Warfarin was administered to 23 patients with isolated CAE and associated varying degrees of myocardial ischemia	Significant decrease of the incidence of unstable angina, positive exercise EKG, and the silent ischemia on EKG Holter monitor
Grigorov [26]./2009	Observational study	Warfarin was administered for life to 15 patients with CAE class I, II, and III	Zero percent mortality rate and low percentage of adverse events for the 4.2-year follow-up period
Doi et al. [24]./2017	Observational study	Warfarin was administered to 19 patients with CAE and MACE at discharge	No recurrence of MACE in patients who took warfarin and achieved the %TTR (≥60%)
Pranata et al. [20]./2019	Systematic review of case reports	Warfarin was administered to eight patients with CAE and ACS	No recurrence of ACS in patients who took warfarin after a mean of 8.4 months follow-up period

Lipid-lowering medications’ role and their relationship to the pathogenesis

CAE has always been thought of as a variant and a result of coronary atherosclerosis [4,7]. Therefore, lipid-lowering medications such as statins were considered for primary prevention [19]. Statins exert their cholesterol-lowering effects through inhibition of the rate-limiting enzyme in the synthesis of cholesterol, and 3-hydroxy-3-methylglutaryl-CoA (HMG-CoA) reductase. Moreover, statins can be used in CAE patients due to their anti-inflammatory properties. Statins have been shown to decrease cyclooxygenase-2 and matrix metalloproteinase-9 expression in the human endothelium, leading to anti-inflammatory and anti-angiogenic effects, which protects against the rupture of atherosclerotic plaques [28].

CAE has been shown to have an inflammatory component due to the presence of higher levels of P-selectin, beta-thromboglobulin, and PF4 in patients with CAE. Also, CAE patients have a higher mean platelet volume. So, platelets with their inflammatory characteristics can play a role in the pathogenesis of CAE [17,18]. In addition, a study by Ozbay et al. measured plasma high-sensitivity C-reactive protein (hs-CRP) levels in 40 patients with CAE and compared them to 41 patients with obstructive coronary artery disease. They found significantly higher levels of hs-CRP in patients with CAE (P<0.0001). They also administered statins and ACE inhibitors to all patients and measured again after three months to find significantly decreased levels of plasma hs-CRP in CAE patients (P<0.0001) [29]. Another study by Tengiz et al. found a significant association between matrix metalloproteinase-3 (MMP-3) and aneurysmal coronary artery disease. So, MMP-3 may be implicated in the pathogenesis of CAE due to their proteolytic activities of extracellular matrix proteins [30]. Statins have also been shown to decrease MMP-3 secretion and activities [31].

CAE has been shown to be more prevalent in patients with familial hypercholesterolemia (FH). A study by Sudhir et al. found that CAE is more prevalent in patients with FH than patients with severe coronary atherosclerosis without FH. As a result, the abnormal lipoprotein metabolism could be the culprit behind the increased prevalence of CAE in patients with FH. They also found significantly lower high-density lipoprotein (HDL) cholesterol (P<0.003) levels and less significantly higher low-density lipoprotein (LDL) cholesterol levels (P<0.07) in patients with FH concomitant with CAE [12], supporting the use of statins in patients with CAE. Moreover, plasma exchange in patients with FH combined with conventional lipid-lowering medications has been shown to decrease the progression of atherosclerosis, decrease the dilatation of the ectatic coronary artery, and reduce serum cholesterol levels more effectively [32].

ACE inhibitors' role and their relationship to genetics

ACE inhibitors have been suggested for the management of CAE due to their anti-hypertensive effects [24]. Controlling systemic hypertension may help slow the progression of coronary dilation by decreasing the intramural pressure [3,33]. ACE inhibitors decrease systemic blood pressure by playing a vital role in the renin-angiotensin-aldosterone system by inhibiting the vasoconstrictive actions of angiotensin II [34]. However, there are other mechanisms that support the use of ACE inhibitors in the management of CAE.

ACE DD genotype polymorphism was found to be a risk factor for CAE. In a study by Gulec et al. on 152 patients with CAE, a significant association (P<0.0046) between ACE DD genotype and the presence of CAE was found when compared to the control group. Patients with CAE were also found to have a more significant association between CAE and ACE DD genotype when compared to ACE II/ID genotype with an adjusted odds ratio of 2.16 [95% confidence interval (CI) 1.34 to 3.41, P=0.0027]. Furthermore, the adjusted odds ratio was 2.16 for ACE DD genotype patients versus II genotype (95% CI 1.12 to 4.14, P=0.02). In addition, the association remained significant after the exclusion of 44 ectatic patients without substantial concomitant coronary artery disease [11].

ACE inhibitors, and possibly ARBs were also proposed for CAE treatment due to their anti-inflammatory properties, especially if there are coexisting indications for them [19]. Like statins, ACE inhibitors can be used to manage CAE due to the significant association between CAE and high levels of hs-CRP [29] and MMP-3 [30]. According to an experimental study by Daugherty et al., it was found that the stimulation of the renin-angiotensin system may result in an enhanced inflammatory response in the vessel wall or the activation of matrix metalloproteinases [35].

Beta-blockers and their anti-ischemic effects

Kruger et al. proposed the use of beta-blockers in patients with CAE since their study concluded that myocardial ischemia is dependent on heart rate. Therefore, beta-blockers could be beneficial due to their negative chronotropic effects and decrease of myocardial oxygen demand in the absence of vasodilation [36]. Beta-blockers have been shown to improve myocardial metabolism in coronary artery disease. In a study by Jackson et al., 20 patients with angina pectoris were tested by stressing the heart using atrial pacing, and it was found that the use of beta-blockers significantly reduced myocardial glucose extraction (P<0.001) and the degree of ST-segment depression (P<0.05). Furthermore, beta-blockers increased myocardial lactate extraction (P<0.02) and pacing time to angina (P<0.01) [37]. In addition, like ACE inhibitors, the use of beta-blockers in CAE can be supported by their anti-hypertensive effects and the role of these effects in decreasing the progression of aneurysmal dilation [3,33,38].

However, the use of beta-blockers in the management of CAE is still a matter of debate. Even though they can decrease the myocardial oxygen demand, they can lead to vasospasm [39]. So, the prescription rate of beta-blockers in these patients was found to be around 50% [24]. Sorrel et al. also argued against the use of beta-blockers as they may increase the risk of coronary vasospasm and lead to an unopposed alpha receptors’ stimulation [25].

Calcium (Ca) channel blockers for prevention of complications

Sorrel et al. were the first to propose Ca Channel blockers for the management of CAE along with aspirin and warfarin. They based their recommendation on case reports and the high association between CAE and the incidence of clinical complications such as coronary thrombus formation and vasospasms. They attributed the use of Ca channel blockers to their anti-spasm effects [25]. Ca channel blockers can also be used due to their anti-hypertensive effects similar to beta-blockers and ACE inhibitors [3,33,38].

In addition, Ca channel blockers can be used for their effects on coronary blood flow. It has been established that CAE is associated with coronary slow flow [15]. A study by Ozcan et al. showed the effects of diltiazem, a nondihydropyridine Ca channel blocker, on the coronary slow flow dynamics and the myocardial perfusion at both epicardial and tissue levels. In 60 patients with isolated CAE, 5mg of diltiazem significantly improved the thrombolysis in myocardial infarction flow grade (P<0.001), thrombolysis in myocardial infarction (TIMI) frame count (TFC) (P<0.001), and the myocardial blush grade (P=0.02) compared to normal saline [40]. Dihydropyridine Ca channel blockers can also be used as coronary vasodilators, especially since there are no reports of complications with this class of medications [19]. Furthermore, a study by Beltrame et al. demonstrated the effects of mibefradil, a T-type Ca channel blocker, in ameliorating the coronary slow flow phenomenon in 20 patients. This medication significantly improved the TFC (P<0.005). It also significantly reduced the frequency and the length of anginal episodes (P<0.001) and the need to use sublingual nitrate (P<0.01) [41]. Unfortunately, mibefradil was withdrawn from the drug market due to adverse drug-drug interactions and is currently being tested in cancer treatment [42].

Antianginal agents and their role in symptomatic management

Nitrates

Sorrel et al. suggested the possibility of using nitrates in managing CAE, but to do so cautiously so as to not chronically expose the patients to these agents by using the concept of a nitrate-free holiday to avoid tolerance. Nitrates are usually used to treat ischemic heart disease by exerting vasodilator effects in large veins and arteries through the delivery of NO [25,43]. However, Kruger et al. argued against the use of nitrates in CAE and proved that nitroglycerin has no therapeutic effect and that it may also lead to a significant worsening of exercise-induced myocardial ischemia (P<0.001). In their study, they also showed that increasing coronary diameters were significantly correlated with the metabolic extent of myocardial ischemia (P<0.001) and impaired coronary blood flow characteristics such as segmental backflow (P<0.04) [36]. So, physicians should be aware of the effect of nitrates in dilated coronopathy and should consider the diagnosis of CAE in a patient with worsening angina after using nitrates [19].

Trimetazidine

Trimetazidine is an antianginal agent that works by increasing intracellular adenosine triphosphate levels and adenosine levels, preventing oxygen-free radical-induced ischemic injury, and changing cardiac energy production from fatty acid oxidation to glucose utilization. The rise of adenosine levels results in myocardial preconditioning, increasing cell tolerance to ischemia [44]. It was found that trimetazidine can have anti-ischemic effects without significantly affecting the heart rate, blood pressure, or the heart rate blood pressure product. It also does not alter the coronary blood flow or oxygen consumption [45]. In the study by Dogan et al., they investigated the effects of trimetazidine on exercise performance in patients with CAE. They found that trimetazidine significantly lowered the rate and extent of ST-segment depressions (P<0.01) in patients with a positive exercise stress test. It also had a significant effect on increasing the cardiac workload (P<0.01) and the exercise duration (P=0.04). Therefore, it could be used to improve exercise tolerance in patients with CAE [44].

Indications of interventional management

Besides the pharmacologic management of CAE, there are other invasive revascularization options such as percutaneous coronary intervention and coronary artery bypass graft [46]. The types, challenges, and outcomes of different interventional strategies have been reviewed extensively in other articles [47,48]. These invasive options are usually indicated in CAE patients with concomitant obstructive lesions or patients exhibiting symptoms or signs of myocardial ischemia despite adequate pharmacologic treatment. However, patients with isolated CAE can be managed with optimal medical therapy alone [46,49]. Thrombus formation is another complication that may happen in CAE. In addition to usual interventional strategies, Tanabe et al. successfully used pulse-spray thrombolysis to treat a thrombotic occlusion of an ectatic right coronary artery in two patients [50].

Limitations

While we were searching for material for this review, there was one limiting factor. Our data was primarily obtained from free access articles only. Therefore, some articles of closed access may have been missed.

## Conclusions

The goal of this review is to shed light on the condition of CAE and its pharmacologic management for clinicians and researchers. To date, there is no consensus regarding the optimal therapeutic regimen for CAE. All of the available studies have limitations such as non-randomization, the absence of a control group, or a small number of patients. It is considerably challenging to conduct large randomized clinical trials because of the rareness of this condition.

According to the available evidence, the management should be tailored to individual patients depending on the presence of isolated CAE or CAE-concomitant obstructive coronary disease. Since CAE represents a form of atherosclerotic coronary artery disease, aggressive risk factor modification for primary prevention using aspirin and statin is deemed necessary in all patients. Secondary prevention using anticoagulation and Ca channel blockers may be needed in some complicated cases. Using beta-blockers and trimetazidine can be employed to manage symptomatic patients suffering from angina. In patients with CAE with concomitant hypertension, ACE inhibitors should be the first line of management due to their evident role in pathogenesis.

There is an unmet need to conduct more studies to test the effectiveness of modern antiplatelets and anticoagulants in patients with CAE. Furthermore, randomized controlled clinical trials are needed to determine whether it is necessary to use dual antiplatelet therapy or the combination of an antiplatelet and an anticoagulant to prevent complications in patients with isolated CAE.
